# Display of multiple proteins on engineered canine parvovirus-like particles expressed in cultured silkworm cells and silkworm larvae

**DOI:** 10.3389/fbioe.2023.1096363

**Published:** 2023-02-16

**Authors:** Jian Xu, Tomofumi Sekiguchi, Jirayu Boonyakida, Tatsuya Kato, Enoch Y. Park

**Affiliations:** ^1^ Laboratory of Biotechnology, Green Chemistry Research Division, Research Institute of Green Science and Technology, Shizuoka University, Shizuoka, Japan; ^2^ Department of Agriculture, Graduate School of Integrated Science and Technology, Shizuoka University, Shizuoka, Japan; ^3^ Department of Bioscience, Graduate School of Science and Technology, Shizuoka University, Shizuoka, Japan

**Keywords:** canine parvovirus, protein display, silkworm, SnoopTag, SpyTag, vaccine conceptualization

## Abstract

Recent progress has been made dramatically in decorating virus-like particles (VLPs) on the surface or inside with functional molecules, such as antigens or nucleic acids. However, it is still challenging to display multiple antigens on the surface of VLP to meet the requirement as a practical vaccine candidate. Herein this study, we focus on the expression and engineering of the capsid protein VP2 of canine parvovirus for VLP display in the silkworm-expression system. The chemistry of the SpyTag/SpyCatcher (SpT/SpC) and SnoopTag/SnoopCatcher (SnT/SnC) are efficient protein covalent ligation systems to modify VP2 genetically, where SpyTag/SnoopTag are inserted into the N-terminus or two distinct loop regions (Lx and L2) of VP2. The SpC-EGFP and SnC-mCherry are employed as model proteins to evaluate their binding and display on six SnT/SnC-modified VP2 variants. From a series of protein binding assays between indicated protein partners, we showed that the VP2 variant with SpT inserted at the L2 region significantly enhanced VLP display to 80% compared to 5.4% from N-terminal SpT-fused VP2-derived VLPs. In contrast, the VP2 variant with SpT at the Lx region failed to form VLPs. Moreover, the SpT (Lx)/SnT (L2) double-engineered chimeric VP2 variants showed covalent conjugation capacity to both SpC/SnC protein partners. The orthogonal ligations between those binding partners were confirmed by both mixing purified proteins and co-infecting cultured silkworm cells or larvae with desired recombinant viruses. Our results indicate that a convenient VLP display platform was successfully developed for multiple antigen displays on demand. Further verifications can be performed to assess its capacity for displaying desirable antigens and inducing a robust immune response to targeted pathogens.

## 1 Introduction

Infectious diseases caused by pathogenic bacteria, viruses, and fungi are long-standing problems for animals and humans, especially those that cause a global pandemic and severe lethality, such as COVID-19 (Kwok et al., 2021; Baker et al., 2022). Besides the social restrictions to lower the spread rate among individuals, immunization with an effective vaccine might be the only ultimate weapon to fight against the pathogens (Micoli et al., 2021; Pollard and Bijker, 2021). To date, there are several main types of vaccines developed, including inactivated, live-attenuated, toxoid, recombinant subunit, messenger RNA (mRNA), and viral vector vaccines (Pollard and Bijker, 2021; Tariq et al., 2021; Mohsen and Bachmann, 2022). Among those types, virus-like particles (VLPs) comprising only one or more structural components and lacking viral genetic materials of an infectious virus are one of the emerging and promising vaccines on the market. Since VLP exists in a virus-mimicking form, those self-assembled nanoscale structures as vaccines offer advantages over traditional approaches, such as efficient immune responses, safety, and adjuvant-free in some cases (Nooraei et al., 2021; Mohsen and Bachmann, 2022). To our knowledge, there have already been some licensed VLP vaccines against HBV (Recombivax HB^®^ and Engerix-B^®^), HPV (Gardasil^®^ and Cervarix^®^), HEV (Hecolin^®^), and malaria (Mosquirix™), and some others are under laboratory research or the clinical trial stages (Tariq et al., 2021).

Two directions of VLP research as vaccine candidates have been continuously focused on in recent decades. One is the VLP production from viral structural proteins in existing protein expression systems, either prokaryotic (bacteria) or eukaryotic (yeast, insect, plant, and mammalian cells) platforms (Nooraei et al., 2021; Tariq et al., 2021). Optimizations such as codons, fusion tags, promoters, and hosts are usually carried out to improve the yield, quality, or cost of expressed proteins of interest (POIs) for VLP formations (Fuenmayor et al., 2017; Minkner and Park, 2018; Nooraei et al., 2021; Mohsen and Bachmann, 2022; Travieso et al., 2022). It is reasonable to screen an appropriate host and protein expression system for better production of POIs with proper posttranslational modifications, e.g., glycosylation and phosphorylation, which are essential for protein solubility, stability, functions, and antigenicity (Moremen et al., 2012; Xu and Ng, 2015; Macek et al., 2019). The baculovirus expression vector system (BEVS) was developed decades ago as a flexible protein production platform using lepidopteran cell lines, larvae, or pupa in laboratory and industrial applications (Kato et al., 2010; Minkner and Park, 2018; Hong et al., 2022). The insect cells, such as *Spodoptera frugiperda* Sf9 and *Bombyx mori* Bm5 cells, are convenient for expressing multiple proteins or protein complexes by co-infection with different recombinant baculoviruses. Now, many POIs have been produced in BEVS (Kato et al., 2010; Minkner and Park, 2018; Hong et al., 2022), some of them, either in the form of subunit (such as Flublok^®^ for influenza virus and Porcilis^®^ Pesti for swine fever) or VLP (such as Cervarix^®^ for human papillomavirus and Porcilis^®^ PCV for Porcine circovirus type 2) were already licensed as vaccines to fight against mammalian pathogens (Felberbaum, 2015; Hong et al., 2022). Compared to the conventional insect cell-based BEVS, the expression platform using silkworm larvae or pupae, *Bombyx mori* (silkworm-BEVS) has been well-established for better protein productions since the first trial for producing human *α*-interferon (Maeda et al., 1985). In scale-up of production, it is convenient to use silkworm-BEVS since a stable and cost-effective rearing method for domestic has already been developed in many Asian contourites (Kato et al., 2010). Moreover, it is rational to consider that each genetically homogenized silkworm can be considered an independent bioreactor, and individual differences in the productivity of recombinant POIs are less likely to occur (Yamashita et al., 2017; Masuda et al., 2022; Xu et al., 2022). However, systematic manufacturing standards and regulations, including Good Manufacturing Practice (GMP), are still under construction for the silkworm-BEVS system to meet the commercial level like well-developed protein production platforms, such as *E. coli*, yeast, and CHO cells (Tomita, 2018).

Another trend in VLPs is to modify the DNA or amino acid sequences genetically or chemically, usually structure-based, to gain new desired functions of VLP as nanoscale delivery vehicles, *e.g.*, surface display of a specific antigen or encapsulation of nucleic acids aiming for antigen expressions *in vivo*. Following the 3Ds (Design, Delivery, and Dynamics) in VLP-based vaccines, VLPs displaying desirable antigens are fascinating strategies to transform available VLPs to the next level for various purposes (Mohsen et al., 2020; Nooraei et al., 2021; Pollard and Bijker, 2021; Tariq et al., 2021). Recently, the covalent peptide ligation technology has been developed to be suitable for protein decorations *in vitro* or *in vivo*, which is also promising for surface display for VLPs in a plug-and-display manner (Brune et al., 2016; Brune et al., 2017; Brune and Howarth, 2018; Bruun et al., 2018; Marini et al., 2019). Two independent protein chemistry, SpyTag/SpyCatcher (SpT/SpC) and SnoopTag/SnoopCatcher (SnT/SnC), derived from *Streptococcus pyogenes* and *Streptococcus pneumoniae*, respectively, have been used as protein tags for specific and robust protein binding, between Lysine (Lys) and Aspartic acid (Asp) or Lys and Asparagine (Asn) (Zakeri et al., 2012; Veggiani et al., 2016; Wong et al., 2020). The specificity and robustness of SpT/SpC and SnT/SnC for programmable protein ligation and VLP display have been validated in various protein expression systems, including *Escherichia coli*, *Bacillus subtilis* 168, yeast, mammalian cells, insect cells and larvae, plant, and cell-free systems (Veggiani et al., 2016; Wang et al., 2016; Kajiwara et al., 2021; Stander et al., 2021; Gallus et al., 2022; Ye et al., 2022). Considerable immune responses have been obtained in several reported experimental models when immunized with those synthetic VLPs displaying single or dual SpT/SnT-decorated antigens (Liu et al., 2014; Brune et al., 2016; Leneghan et al., 2017; Bruun et al., 2018; Zhang et al., 2020; Lampinen et al., 2021).

Previously, we successfully generated SpT/SpC-based BEVS (SpyBEVS), demonstrating efficient protein binding activity between SpT and SpC partners either in purified proteins *in vitro* or co-expressed forms in silkworm larvae *in vivo via* the viral co-infection (Xu et al., 2019). To exploit the potential of SpyBEVS for plug-and-playable VLPs, we choose the capsid protein VP2 of canine parvovirus (CPV, non-enveloped T = 1 icosahedron, 60 subunits, 26 nm in diameter) as a VLP model. The CPV-LP has been frequently expressed in insect cells or silkworm pupae with the BEVS, showing high immunogenicity (Hurtado et al., 1996; Yuan and Parrish, 2001; Feng et al., 2011; Elia et al., 2012; Feng et al., 2014; Jin et al., 2016; Chang et al., 2020). Moreover, the three-dimension structural information of CPV was available (PDB: 4QYK), and four loop regions (Loop 1–Loop 4) were identified and studied for their essentiality of VLP formations (Tsao et al., 1991; Hurtado et al., 1996). Loop 2 (R216-G235) has been proven not essential for VLP assembly and thus could be further engineered for antigen/epitope display purposes (Brown et al., 1994; Hurtado et al., 1996; Feng et al., 2011; Xu et al., 2014).

In the current study, we genetically engineered a series of chimeric VP2 protein variants inserted with SpT at different locations, showing that VP2-SpT-Loop2 variant-derived VLPs significantly showed enhanced display abundance of a SpC-tagged green fluorescent as a model protein. The two candidate loop domains are further introduced with SpT or SnC for orthogonal multiple antigen surface displays. The intact VLP was engineered using SpT/SnT double-tagged VP2 variant and ligated orthogonally among binding partners, *in vivo* and *in vitro.* We herein successfully developed a flexible VLP display platform for multiple antigen surface display on demand, paving the way to further verifications of antigens displayed VLPs as vaccine candidates in various animal-pathogen models.

## 2 Materials and methods

### 2.1 Silkworm larvae and cells

The silkworm fourth instar larvae were purchased from Ehime Sansyu (Ehime, Japan) and reared with an artificial diet (Silkmate S2, Nosan, Japan) under a controlled environment (25°C, 65% ± 5% relative humidity) for 4∼5 days to reach fifth instar larvae for baculovirus infections. The cultured silkworm Bm5 cells were routinely passaged in Sf-900II medium (ThermoFisher Scientific Tokyo, Japan) supplemented with 10% fetal bovine serum (Gibco, Tokyo, Japan) and 1% antibiotic-antimycotic (ThermoFisher Scientific, Tokyo, Japan) at 27°C.

### 2.2 Plasmid and baculovirus constructions containing SpT-SpC or SnT-SnC

The synthesized VP2 gene of Canine parvovirus (NC_001539.1) by Genewiz (Suzhou, China) was amplified *via* polymerized chain reaction (PCR) using KOD-Plus-Neo DNA polymerase (Toyobo, Tokyo, Japan). The VP2 gene was subcloned to pFastBac1-SpT/SpC (Spy002 version, SpyTag002/SpyCatcher002) (Keeble et al., 2017) plasmids which were generated in our previous study (Xu et al., 2019). The variants for VP2 with inserted SpT or SnT (Genewiz, Suzhou, China) at Loop 2 (S226_G227) or Loop X (T391_G392) regions were constructed by inverse PCR using phosphorylated primers (T4 Polynucleotide Kinase, NEB, MA, United States) listed in Table 1. The transfer pFastBac plasmids, pFastbac-SpC-EGFP (Xu et al., 2019), pFastBac-SnC-mCherry, pFastBac-SpC-VP2, pFastBac-noSpT-VP2, pFastBac-NSpT-VP2, pFastBac-SpTL2-VP2, pFastBac-SpTLx-VP2, pFastBac-SpT_L2_SnT_Lx_-VP2, pFastBac-SnT_L2_SpT_Lx_-VP2, and pFastBac-∆StrepTag-SpT_L2_SnT_Lx_-VP2 for producing bacmid DNA were transformed into *E. coli* BmDH10Bac cells (Motohashi et al., 2005). This study used double fusion tags, His6-StrepTagII (HS) or FLAG-StrepTagII (FS), to facilitate the detection and purification among different constructs (Table 2). All the sequences were confirmed directly by DNA sequencing. The corresponding bacmid was subsequently transfected into either cultured Bm5 cells or silkworm fifth instar larvae using 1,2-dimyristyloxypropyl-3-dimethyl-hydroxy ethyl ammonium bromide and cholesterol (DMRIE-C, ThermoFisher Scientific K. K., Tokyo, Japan) transfection reagents (2 μg/1 μL bacmid DNA) or chitosan/bacmid system to obtain recombinant baculoviruses according to the protocols established previously (Kato et al., 2016; Xu et al., 2019). The series infection method obtained the stock of high titer viruses employed for infecting silkworm cells and larvae.

**TABLE 1:** List of primers used in this study.

Name	Sequence (5′–3′)
VP2-Fw	Tcc​gac​ggt​gcc​gtg​caa​cc
VP2-Rv	Ggg​ctg​tga​gta​cag​ttt​acg​agg​ggc​ga
TEV-Rv	Acc​ttg​gaa​gta​taa​att​ctc​tga​tc
L2SpT-Fw	Gtg​gac​gct​tac​aag​cgc​tac​aaaggc​ggt​ggc​tca**ggt**acc​ccc​act​aac​atc​tac​cac​ggc​acc
L2SpT-Rv	Cat​cac​gat​tgt​agg​cacacc​aga​acc​aga​tga​acc**gga**ggt​gcc​ggt​gtg​gga​ggg​gat​cag​agt
LxSpT-Fw	Gtg​gac​gct​tac​aag​cgc​tac​aaaggc​ggt​ggc​tca**ggc**gag​act​ccc​gaa​cgc​ttc​act​tac​atc
LxSpT-Rv	Cat​cac​gat​tgt​agg​cacacc​aga​acc​aga​tga​acc**ggt**agt​ggt​ggt​ctt​ctg​gcc​gtg​ttg​gcg
L2SnT-Fw	Aag​ctg​ggt​gac​atc​gag​ttc​atc​aag​gtg​aac​aagggt​ggc​ggc​agt**ggt**acc​ccc​act​aac​atc​tac​cac​ggc​acc
L2SnT-Rv	Acc​gga​ccc​aga​act​acc**gga**ggt​gcc​ggt​gtg​gga​ggg​gat​cag​agt
LxSnT-Fw	Aag​ctg​ggt​gac​atc​gag​ttc​atc​aag​gtg​aac​aagggt​ggc**ggc**agt​ggc​gag​act​ccc​gaa​cgc​ttc​act​tac​atc
LxSnT-Rv	Acc​gga​ccc​aga​act​acc**ggt**agt​ggt​ggt​ctt​ctg​gcc​gtg​ttg​gcg
SnC-Fw	Aag​cct​ctg​cgc​ggt​gct​gtg​ttc
SnC-Rv	Gag​ctc​acc​aga​acc​acc​aga​acc​aga​tga​acc​ctt​agg​agg​gat​agg​ctc​gtt​ggt​g

Underlined: SpT or SnT sequences; **bold**: L2 or Lx sites.

**TABLE 2:** Predicted molecular weight for the proteins used in this study.

Type	Name	Description	Tags	MW^a^ (kDa)	Labeling	Location
**Free proteins**	SpC-VP2	SpC-fused VP2	His6-Strep (HS)	81.3	N.A.	Figure 1
	(N)SpT-VP2	N-terminal SpT-fused VP2	FLAG-Strep (FS)	70.8	N.A.	Figures 1–3
	noSpT-VP2	VP2 without SpT fusion	HS	68.1	N.A.	Figures 2, 3
	SpTL2-VP2	Loop 2 SpT-fused VP2	FS	70.5	N.A.	Figures 2, 3
	SpTLx-VP2	Loop X SpT-fused VP2	FS	70.5	N.A.	Figures 2, 3
	SpC-EGFP	SpC-fused EGFP	HS	43.8	#1	Figures 1–4
	SnC-mCherry	SnC-fused mCherry	FS	43.5	#2	Figure 4
	SpTL2-SnTLx	Loop 2 SpT-fused, Loop X SnT-fused VP2	HS	72.7	#3	Figure 4
	SnTL2-SpTLx	Loop 2 SnT-fused, Loop X SpT-fused VP2	HS	72.7	#4	Figure 4
	∆StrepTag-SnTL2-SpTLx	Loop 2 SpT-fused, Loop X SnT-fused VP2 without StrepTag	Deleted	69.1	#5	Figure 4
**Protein conjugation**	(N)SpT-VP2	SpT/SpC ligation	FS, HS	114.6	N.A.	Figures 1D, E; Figure 2D; Figures 3C–E
	::SpC-EGFP					
	SpTL2-VP2	SpT/SpC ligation	FS, HS	114.3	N.A.	Figure 2D; Figures 3C, D, F
	::SpC-EGFP					
	SpTLx-VP2	SpT/SpC ligation	FS, HS	114.3	N.A.	Figure 2D; Figure 3C
	::SpC-EGFP					
	SnTL2-SpTLx	SpT/SpC ligation	HS	116.5	#1, 4	Figure 4B
	::SpC-EGFP					
	SpTL2-SnTLx	SnT/SnC ligation	HS, FS	116.2	#2, 3	Figure 4B
	::SnC-mCherry					
	SpTL2-SnTLx	SpT/SpC ligation	HS	116.5	#1, 3	Figure 4B
	::SpC-EGFP					
	SnTL2-SpTLx	SnT/SnC ligation	HS, FS	116.2	#2, 4	Figure 4B
	::SnC-mCherry					
	∆StrepTag-SnTL2-SpTLx	SpT/SpC ligation	∆, HS	112.9	#1, 5	Figures 4C, D
	::SpC-EGFP					
	∆StrepTag-SnTL2-SpTLx	SnT/SnC ligation	∆, FS	112.6	#2, 5	Figure 4C
	::SnC-mCherry					
	SpTL2-SnTLx	SpT/SpC and SnT/SnC ligation	HS, HS, FS	160	#1, 2, 3	Figure 4B
	::SpC-EGFP					
	::SnC-mCherry					
	SnTL2-SpTLx	SpT/SpC and SnT/SnC ligation	HS, HS, FS	160	#1, 2, 4	Figure 4B
	::SpC-EGFP					
	::SnC-mCherry					
	∆StrepTag-SnTL2-SpTLx	SpT/SpC and SnT/SnC ligation	∆, HS, FS	156.4	#1, 2, 5	Figure 4C
	::SpC-EGFP					
	::SnC-mCherry					
	(∆StrepTag-SnTL2-SpTLx::SpC-EGFP)	SnT/SnC ligation	(∆, HS), FS	156.4	#1, 2, 5	Figure 4E
	::SnC-mCherry					

^a^ Note that the coupling products from protein conjugation show apparently larger MWs, than predictions (*italic*), possibly because of the resulting protein conformation, which significantly affects the migration in SDS-PAGE gels (Schoene et al., 2014; Alam et al., 2019; Zhou et al., 2020; Lemetti et al., 2022).

### 2.3 Bioinformatics analysis and surface loop region assignment of the VP2 protein

To obtain suitable candidates for protein display on VP2-derived VLPs, the three-dimension structural information was adopted (PDB: 4QYK) and visualized in the CLC Sequence Viewer (ver8.0, Qiagen). As already depicted in several previous reports, four loop regions, Loop 1 (aa V84–D99, at the top of protrusion: V92, N93), Loop 2 (R216–G235; H222–T228), Loop 3 (P295–I306; A300–F303), and Loop 4 (Y409–Y444; N421–N428, T433–N443), have been studied, among which aa 211–240 has been suggested to be not essential for VLP formation (Tsao et al., 1991; Hurtado et al., 1996; Xu et al., 2014). In this study, except for a reported Loop 2 (S226_G227, L2) surface loop region (Rueda et al., 1999), another simulated loop candidate (T391_G392, LoopX, Lx) was marked to the outer surface of VP2 protein (Xie and Chapman, 1996) and VP2-derived VLPs and further used for inserting SpT/SnT.

### 2.4 Expression and purification of POIs from silkworm fat body

Expression and purification of all proteins in cultured Bm5 cells or silkworm larvae were performed according to our previous reports (Xu et al., 2019; Xu et al., 2022). Briefly, the fat body from silkworm larvae at 5 days post-infection (dpi) was resuspended and sonicated in a lysis buffer (100 mM Tris-HCl pH 8.4, 0.15 M NaCl, 1 mM EDTA, 0.1% NP-40, 1 × proteinase inhibitor (Roche, Tokyo, Japan)). The clear supernatants were then analyzed by sodium dodecyl sulfate-polyacrylamide gel electrophoresis (SDS-PAGE), followed by either Coomassie brilliant blue (CBB) staining or western blotting. Protein samples were separated on 12% SDS-PAGE gel and then transferred onto a polyvinylidene fluoride (PVDF) membrane by a semidry transfer cell (Bio-Rad, Hercules, CA, United States). Subsequently, the membrane was blocked for 1 h in TBST buffer (0.1%v/v Tween 20 in Tris-buffered saline) containing 5% w/v skimmed milk (Wako, Tokyo, Japan) at room temperature. The washed membrane was then incubated with anti-StrepTagII primary antibody (1:5,000, anti-mouse, QIAGEN, Hilden, Germany), followed by incubation with horseradish peroxidase (HRP)-conjugated secondary antibody (1:10,000, GE Healthcare, Piscataway, NJ, United States). The final development was carried out using Immobilon western chemiluminescence HRP substrate (Merck Millipore, Darmstadt, Germany), which was visualized by VersaDoc MP imaging systems (Bio-Rad) for verifying expressed proteins.

After the expression was validated, protein purification was performed using fat body lysates from baculovirus-infected silkworm larvae (10∼20). Briefly, the lysates were centrifuged at 8,000 g for 30 min at 4°C to remove the insoluble material, followed by filtration with a 0.45-μm filter (Millipore). The × 10 diluted supernatants in a binding buffer (100 mM Tris–HCl pH 8.4, 0.15 M NaCl, 1 mM EDTA) were applied to a StrepTrap column (QIAGEN, Hilden, Germany). After × 10 washing, the target protein was eluted by a binding buffer containing 2.5 mM desthiobiotin (IBA Lifesciences, Germany). The purified proteins were concentrated by Amicon Ultra-15 (MWCO 30 kDa, Millipore) and buffer-exchanged in PBS buffer as demanded. The final protein concentration for each purified protein in this study was estimated by a BCA protein quantification kit (Bio-rad).

### 2.5 *In vivo* and *in vitro* ligation assay using purified proteins or co-infection approach in cultured silkworm cells and larvae

The recombinant baculoviruses stock was directly employed to infect/co-infect cultured cells in a 6-well plate or injected/co-injected into the silkworm larvae on the third day of the fifth instar. At 4 dpi, cultured cells or fat body tissues from 10 silkworm larvae were collected and lysed in a lysis buffer (100 mM Tris–HCl pH 8.4, 0.15 M NaCl, 1 mM EDTA in 0.1% NP-40) with complete EDTA-free protease inhibitor tablet (1 tablet/100 mL; Roche). The clear supernatant was subjected to SDS-PAGE and western blot to detect specific adducts after *in vivo* ligation in silkworms. The *in vitro* ligation assay between purified SpT/SpC- or SnT/SnC-tagged proteins was investigated in the mixture with the corresponding proteins partners under different ratios where a final 20 μL volume was adjusted with PBS buffer. The ligation reaction was performed at room temperature (∼25°C) or 4°C for an indicated time from 1∼24 h in PBS, where the VP2 proteins tested (NSp-VP2, SpTL2-VP2, SnTL2-SpTLx, and ∆StrepTag-SnTL2-SpTLx) are self-assembled into VLPs. The reaction samples mixed with 1 × SDS sampling buffer were heated in 99°C for 10 min before the SDS-PAGE assay to denature proteins and disturb VLPs. The ligation efficiency was estimated by investigating the formed adduct between binding partners from three independent CBB-stained SDS-PAGE.

### 2.6 Transmission electron microscopy (TEM) and dynamic light scattering (DLS) analysis

The purified and concentrated proteins from VP variants were subjected to TEM to confirm the VLP formation and morphology following our previous publications (Suhaimi et al., 2019; Utomo et al., 2022). Briefly, a 20 μL protein solution drop was loaded onto the surface of a carbon film-supported copper grid (200 mesh, Nisshin Em Co. Ltd., Tokyo, Japan) and incubated at room temperature for 30 s. The grid was three-time washed with PBS and negatively stained with phosphotungstic acid (2% v/v). The immune-TEM was performed to investigate the possible VLP surface display in loop regions. The VLP sample on the grid was firstly blocked in 2% bovine serum albumin (BSA) for approximately 5 min, followed by wash and incubation with primary antibody (anti-His6, 1:30 in PBS, MBL, Nagoya, Japan) for 1 h in room temperature. After washing with PBS, the grid was washed six times and incubated with a secondary antibody of goat anti-rat IgG-conjugated 12 nm gold beads (1:50 in PBS, FUJI- FILM Wako Pure Chemical) for 1 h. Subsequently, the PBS-washed grid was negatively stained with phosphotungstic acid (2% v/v), which was visualized in the TEM apparatus (JEM-2100F, JEOL, Ltd., Tokyo, Japan). The DLS was used to analyze the size distribution of formed VLPs which was measured by the Zetasizer Nano series (Malvern Inst. Ltd., Malvern, United Kingdom).

## 3 Results

### 3.1 Terminal SpyTag-engineered capsid VP2 demonstrates low VLP display abundancy

**FIGURE 1:**
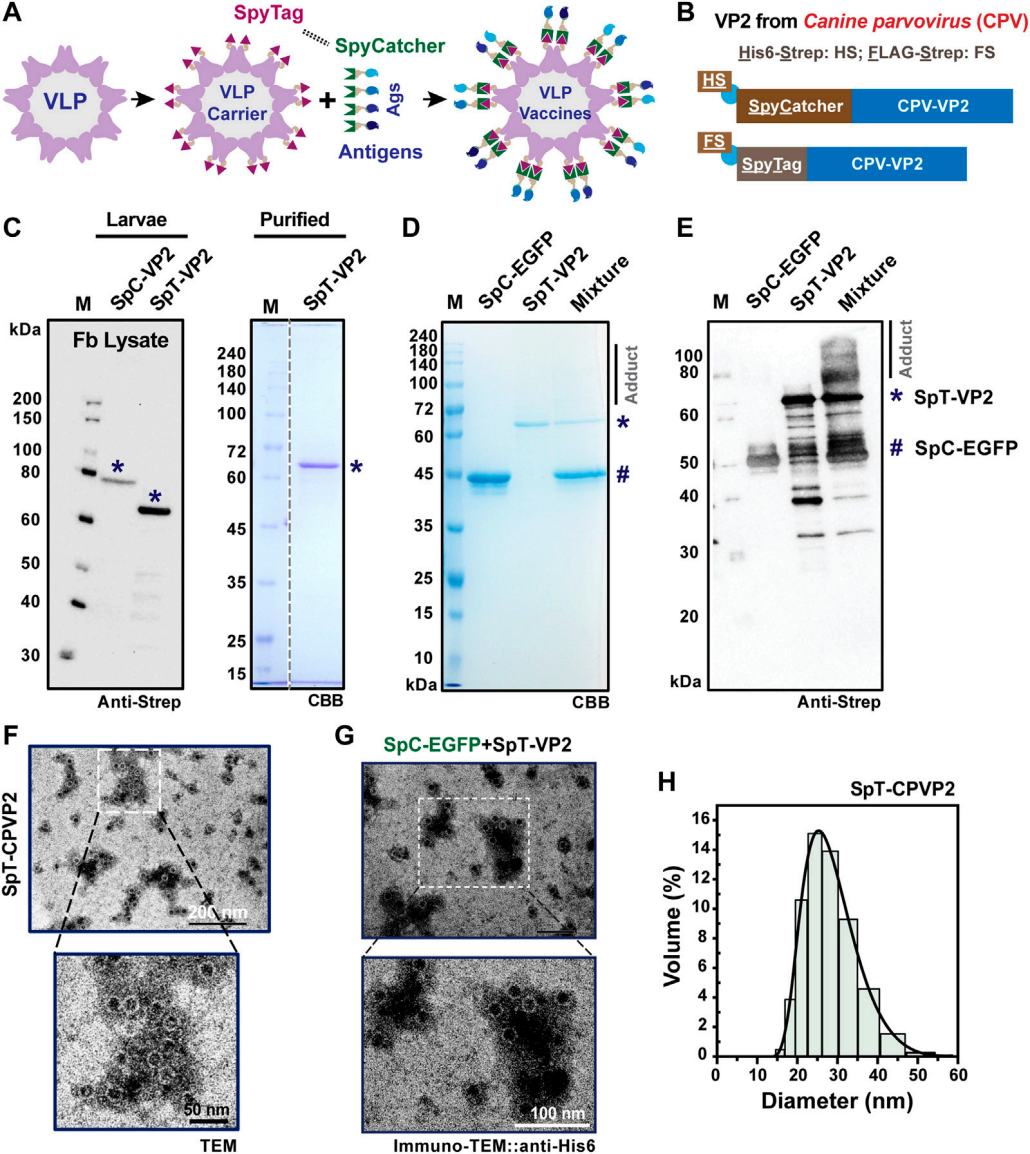
SpyCatcher-SpyTag-mediated protein-protein ligation in VP2-derived VLP platform. A schematic representation of protein or antigen (Ags) displayed on the VLP surface is shown in **(A)**. SpyCatcher (SpC, His6-StrepII (HS)-tagged) or SpyTag (SpT, FLAG-Strep II (FS)-tagged) was designed to attach at the N-terminus of CPV-VP2 for surface exposure, and the reaction between SpC and SpT **(B)**. The expression of SpC- or SpT-fused VP2 protein was verified in the cultured Bm5 cells **(C)**, indicated by *) by western blot using Strep-tag II antibody. The SpT-VP2 protein was further purified from the fat body of virus-infected silkworm larvae (*). The *in vitro* covalent binding efficiency was performed between purified SpT-VP2 (*) and SpC-EGFP (#) proteins in both CBB **(D)** or western blot **(E)**, anti-Strep). The adduct can be seen clearly in the western blot, although only smear bands were detected in CBB gels. SpT-VP2 can structurally form VLPs with a typical diameter of 20–25 nm as assayed in transmission electron microscopy (TEM) and dynamic light scattering (DLS) **(F,H)**. Surface display of EGFP was observed in immuno-TEM (anti-His6) **(G)**.

The VLP formation of CPV can be obtained *via* the self-assembly of capsid VP2 protein in various protein expression systems, including BEVS with cultured insect Sf9 cells, silkworm Bm5 cells, silkworm larvae, and pupae (Choi et al., 2000; Feng et al., 2014; Jin et al., 2016; Chang et al., 2020). Previous literature has also found that the N-terminal genetically fused GFP can be used as a protein display strategy (Gilbert et al., 2006). Thus, as a continuous effort for our previous SpyBEVS (Xu et al., 2019), we designed several SpT-SpC chemistry constructs at terminal regions to establish protein display on CPV-LPs in silkworm-BEVS (SpyVLP-BEVS), which are illustrated in Figures 1A, B. As shown in Figure 1C, the VP2 with N-terminal SpT or SpC can be expressed in silkworm-BEVS, while SpC-VP2 (81.3 kD) has a lower expression level. We then purified SpT-fused VP2 (SpT-VP2, 70.8 kD) proteins from fat body lysates of recombinant baculovirus-infected silkworm larvae (∼3.5 mg/10 silkworm larvae, Figure 1C). The considerable amount of purified VP2 from silkworm-BEVS offered an excellent platform as a good candidate for further protein engineering.

Subsequently, we performed an *in vitro* binding assay between purified SpC-EGFP (43.8 kD) (Xu et al., 2019) and SpT-VP2 at 4°C overnight. We understand that the efficiency of the protein conjugation between SpC and SpT partners depends mainly on the conformation and location of SpC/SpT since the amidation itself is tolerant even to various harsh conditions (Zakeri et al., 2012; Veggiani et al., 2016; Xu et al., 2019; Wong et al., 2020). Previous results have reported that purified SpT-VP2 monomers expressed from silkworm-BEVS were efficiently self-assembled into VLP forms (Feng et al., 2011; Feng et al., 2014). Although the result from SDS-PAGE (Figure 1D) showed an invisible portion of the adduct after coupling, the result from western blot (Figure 1E) showed that only a tiny portion of SpC-EGFP was ligated to SpT-VP2 (∼115 kD). This means that the N-terminal SpT-tagged VP2-derived VLPs exposed SpT in a limited abundance, possibly due to the improper location and/or structural orientation of the presented SpT on the surface of the assembled VLPs. One can also notice the multiple bands for SpT-VP2 and adducts in Figure 1E, which might be caused by the VP2 protein degradations during the reaction in PBS buffer at room temperature, indicating that proteinase inhibitors may be required to prevent such degradations. The low display level is consistent with a previous study of N-terminal EGFP-fused VP2-derived VLP since only 20% (12 out of 60, N-terminal fused EGFP protrudes through the 5-fold axis cannon structures) display rate was simulated. A 16.7% (10/60) could be experimentally achieved on the VLP surface (Gilbert et al., 2006). Before further modifications of the location of SpT, we confirmed the VLP formation of the self-assembled SpT-VP2 in TEM (Figure 1F) and investigated the possible protein display by immuno-TEM using anti-His6 for SpC-EGFP (Figure 1G). The intact VLP structure and distribution (20–30 nm) were validated (Figure 1H) in this study, consistent with previous studies (Choi et al., 2000; Gilbert et al., 2006). This result provides direct evidence that VP2-derived SpT-SpC partner can be successfully expressed in silkworm-BEVS and be realized as a VLP display system. However, the protein display on SpT-VP2-derived VLP was not sufficiently achieved, as confirmed in Figure 1G, where no gold particles were found on VLPs, indicating that the location of SpT needs further optimization for better surface display.

### 3.2 Rational design of insertion of SpT into two loop regions (S226 and T391) of VP2

**FIGURE 2:**
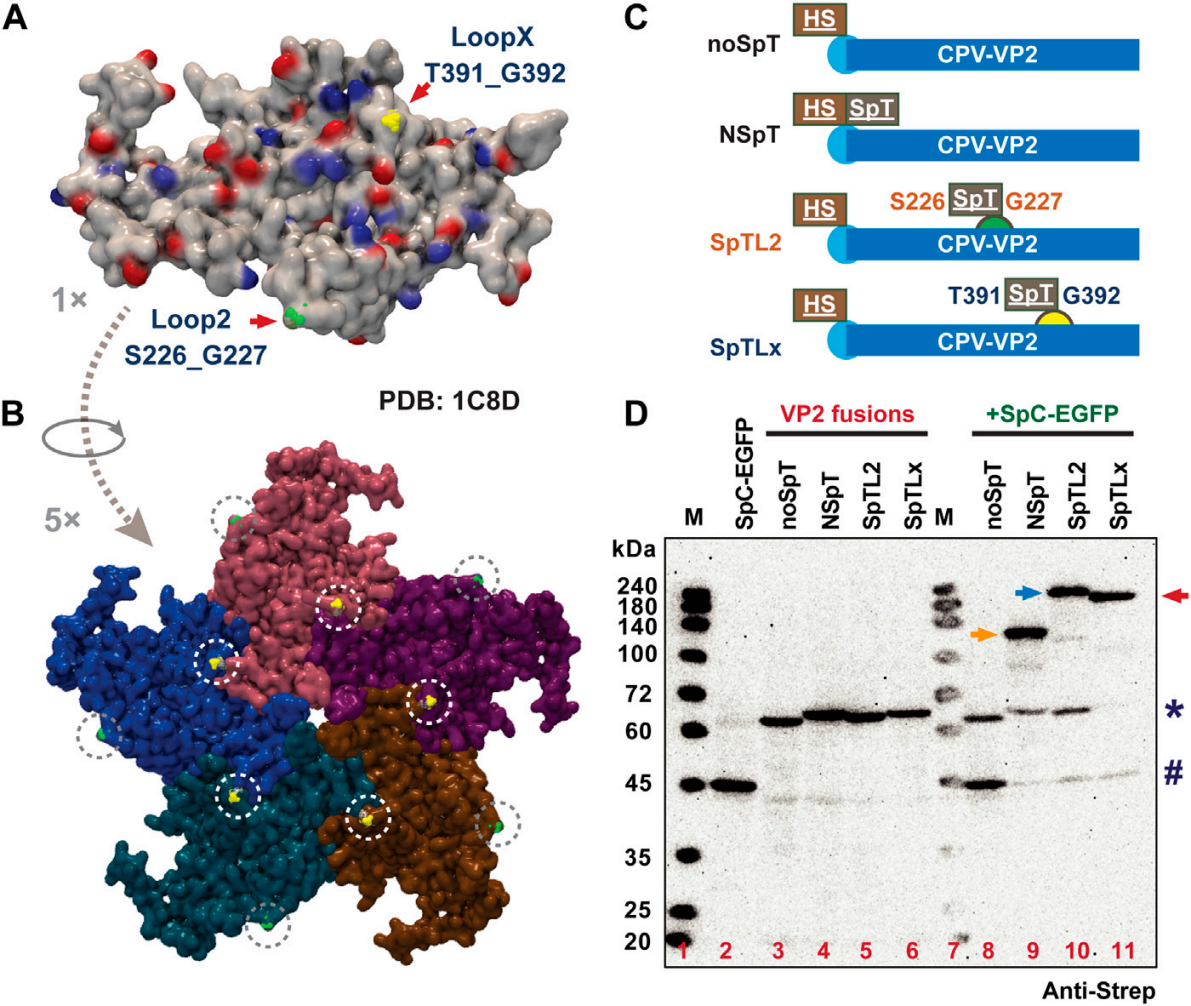
Rational design of SpT exposure site on VP2 structural protein-derived VLPs. The structure of a VP2 protein monomer **(A)** and partial assembly **(B)** were demonstrated (PDB: 4QYK). The two simulated loop regions, S226_G227 and T391_G392, were indicated as Loop 2 (green) and Loop X (yellow) by arrows, which are expected to present on the surface of VLP (60 per VLP). The SpT was designed to insert into different regions of VP2. The variants used in this study were summarized in **(C)** noSpT (No SpT was inserted), NSpT (N-terminus attached), SpTL2 (Loop 2 attached), and SpTLx (Loop X attached). The expression and binding ability between all VP2 SpT-fusions and SpC-EGFP in cultured Bm5 cells were verified by western blot **(D)**, anti-Strep). # and * indicate SpC-EGFP and VP2 variants, respectively. Arrows indicate possible ligated adducts upon co-infection of SpC-SpT partners.

Based on the structure of VP2 (PDB ID: 4QYK) and previous reports (Brown et al., 1994; Hurtado et al., 1996; Feng et al., 2011; Xu et al., 2014), we propose two loop sites, Loop 2 (S226_G227) and Loop X (T391_G392), as candidates for inserting SpT (Xie and Chapman, 1996; Rueda et al., 1999). Theoretically, these two loop regions are supposed to display at a 1: 1 ratio on the protein and assembled VLPs (60 per VLP), as shown in Figure 2A (1 ×, monomer) and **2B** (5 ×, pentamer). Considering the peptide length of SpC (SpyCatcher002, 113 aa) and SpT (SpyTag002, 14 aa) (Keeble et al., 2017; Brune and Howarth, 2018), we propose that the shorter SpT peptide might be suitable to be inserted into the loop region of the VP2 protein without affecting its VLP formation. To test our strategy, we designed a series of chimeric VP2 variants without (noSpT, 68.1 kD) or with SpT at different locations, N-terminus (NSpT, 70.8 kD), Loop 2 (SpTL2, 70.5 kD), and LoopX (SpTLx, 70.5 kD) (Figure 2C). The expression level and correct molecular weight for each construct were verified in Bm5 cells. The adducts from several combinations for co-infection (virus ratio = 1:1) between each VP2 variant and SpC-EGFP were also validated in Figure 2D. As expected, the specific binding activity of each VP2 to SpC-EGFP was nicely achieved, indicating that the ligation occurred efficiently in cultured Bm5 cells. This conjugation reaction was in a relative expression level-dependent manner, where the remaining free SpT-VP2 or SpC-EGFP were significantly decreased in co-infection samples (Lanes 9–11) as compared to that in single infection samples (Lanes 2, 4–6).

### 3.3 Verification of an enhanced surface display in loop2 (S226) SpT-inserted VLPs

**FIGURE 3:**
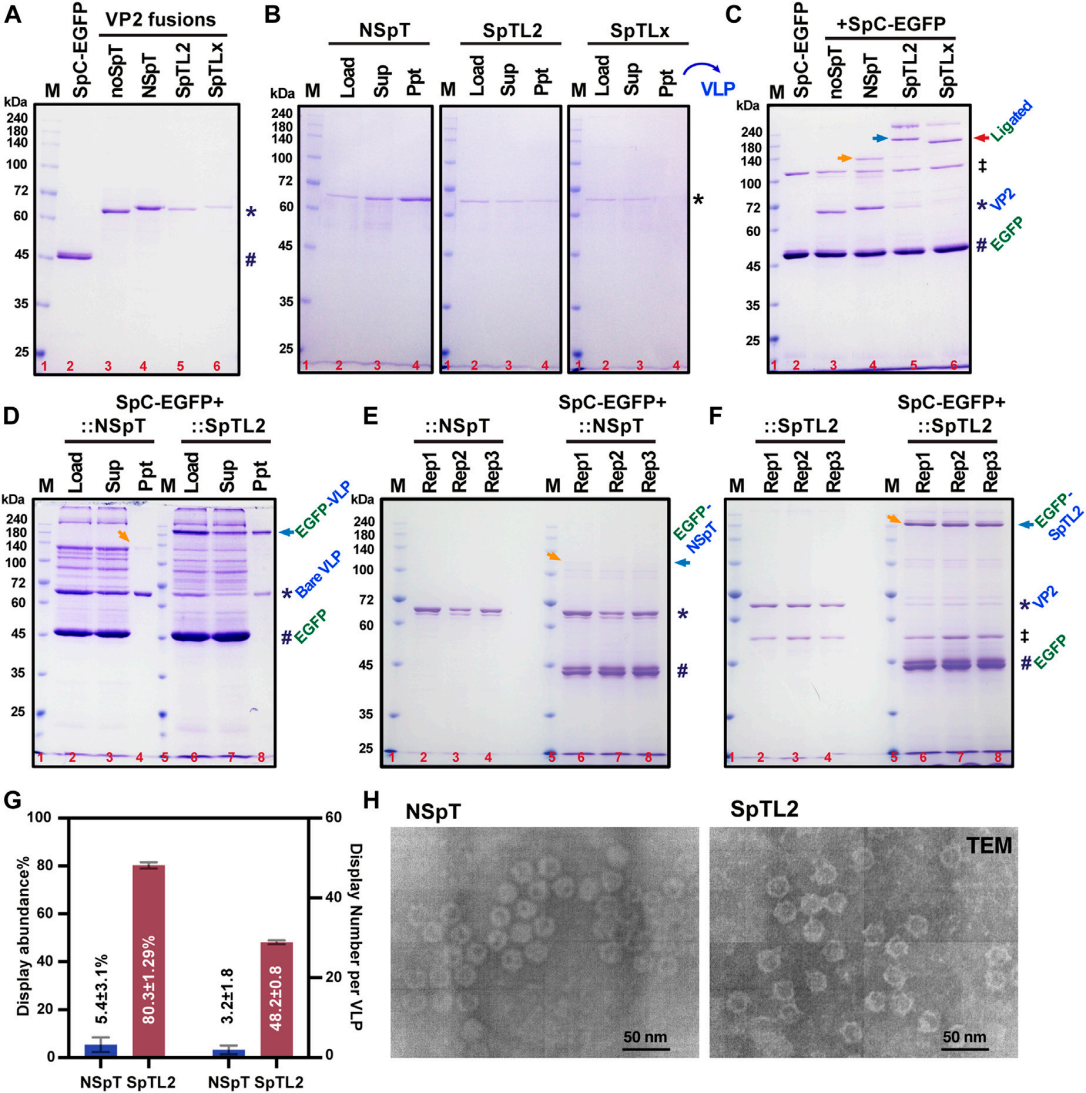
Verification of enhanced protein display in SpT-inserted VP2-derived VLPs. All SpC-EGFP and VP2 without SpT (noSpT) or with SpT at N-terminus (NSpT), Loop2 (SpTL2), and LoopX (SpTLx) were expressed and purified from the fat body of each recombinant baculovirus-infected silkworms **(A)**. VLP formation and purification were performed through ultracentrifugation with a 20% sucrose cushion **(B)**. *In vitro* covalent binding efficiency between SpC (#) and SpT (*) variants was validated using saturated SpC-EGFP (#) proteins as indicated in **(C)**. Ligated adducts were marked with arrows. The double dagger (‡) symbol was used to indicate an impurity from the purification of SpC-EGFP. Reaction products between SpC-EGFP and VP2 variants, NSpT and SpTL2, were further subjected to ultracentrifugation with 20% sucrose solution. VLP products, wither bare VLP (*) and EGFP-decorated VLP (EGFP-VLP, arrow), were shown in CBB **(D)**. The binding assay was repeated three times **(E,F)**, and the precipitations showing the relative amount of EGFP-VLP were statistically analyzed and summarized in **(G)**, which demonstrated an apparent increase of EGFP-displayed VLP from about 5.4% (3 per VLP) to 80% (48 per VLP). The double dagger (‡) symbol was used to indicate an impurity from the purification of SpTL2. The VLP formation for NSpT and SpTL2 variants were validated in TEM **(H)**.

We purified the VP2 variants from silkworm larvae for verifying *in vitro* binding and display assay. As demonstrated in Figure 3A, all purified proteins were obtained in decent amounts (mg scale per 10 silkworms) from only 10 silkworm larvae. However, there was a slight decrease in final yields for two loop variants (∼2.3 mg for SpTL2, ∼1.1 mg for SpTLx), possibly due to the relatively higher loss during Strep-column purification. To confirm whether the purified VP2 proteins could form VLPs correctly and efficiently, we conducted gentle purification of formed VLP from purified VP2 monomers where a standard 20% sucrose cushion was employed (Huhti et al., 2010; Kato et al., 2022). After ultracentrifuge at 122,000 × *g*, the fractions from the supernatant (monomers and nonVLPs) and precipitation (VLPs) were loaded to SDS-PAGE. It was shown in Figure 3B that the NSpT-VP2 and SpTL2-VP2 formed VLP mainly. However, no visible band of VLPs was detected in SpTLx-VP2 samples, indicating some problems for SpTLx-VP2 to form structurally correct VLPs after SpT was inserted into the LoopX region since no intact VLP formation was identified in TEM (Hurtado et al., 1996).

Subsequently, we continue to test if the formed SpT-decorated VLPs could be used as a carrier to further surface display SpC-fused proteins (SpC-EGFP) by *in vitro* mixing the two partners directly in a PBS-buffered solution. As demonstrated in Figure 3C, the specific ligation between SpT proteins (NSpT, SpTL2, and SpTLx) and saturated SpC-EGFP occurred as expected, where no shifted bands were observed under noSpT-VP2 conditions. Since we have already confirmed in Figure 3B that VLP formation is not sufficiently ensured in SpTLx-VP2, the adducts from the mixture of SpTLx-VP2 and SpC-EGFP should come mainly from the SpTLx-VP2 monomer other than formed VLPs. Afterward, we then focused on the SpTL2-VP2-derived VLPs. The purification using 20% sucrose cushion for SpC-EGFP mixed with NSpT or SpTL2 showed a significant increase in the VLP displaying SpC-EGFP in SpTL2 as judged by the ratio of the relative band intensity between bare VLP and EGFP-displaying VLP (EGFP-VLP) (Figure 3D). The display abundance, either in percentage (%) or number per VLP, was estimated from three independent binding assays (Figures 3E, 3F). As depicted in Figure 3G, protein display abundance was improved from 5.43% in NSpT-VP2 to 80.3% in SpTL2-VP2, which is 3–48 per VLP when calculated for the number of proteins. It indicates that the loop-inserted SpT-exposing to the surface of formed VLPs directly contributes to the significant enhancement of protein display on VLPs. The obtained results of the VLP formation for SpTL2-VP2 and the control from NSpT-VP2 validated by TEM are shown in Figure 3H.

### 3.4 SpT-SnT double-decorated VLPs provide a flexible multiple-protein display system

**FIGURE 4:**
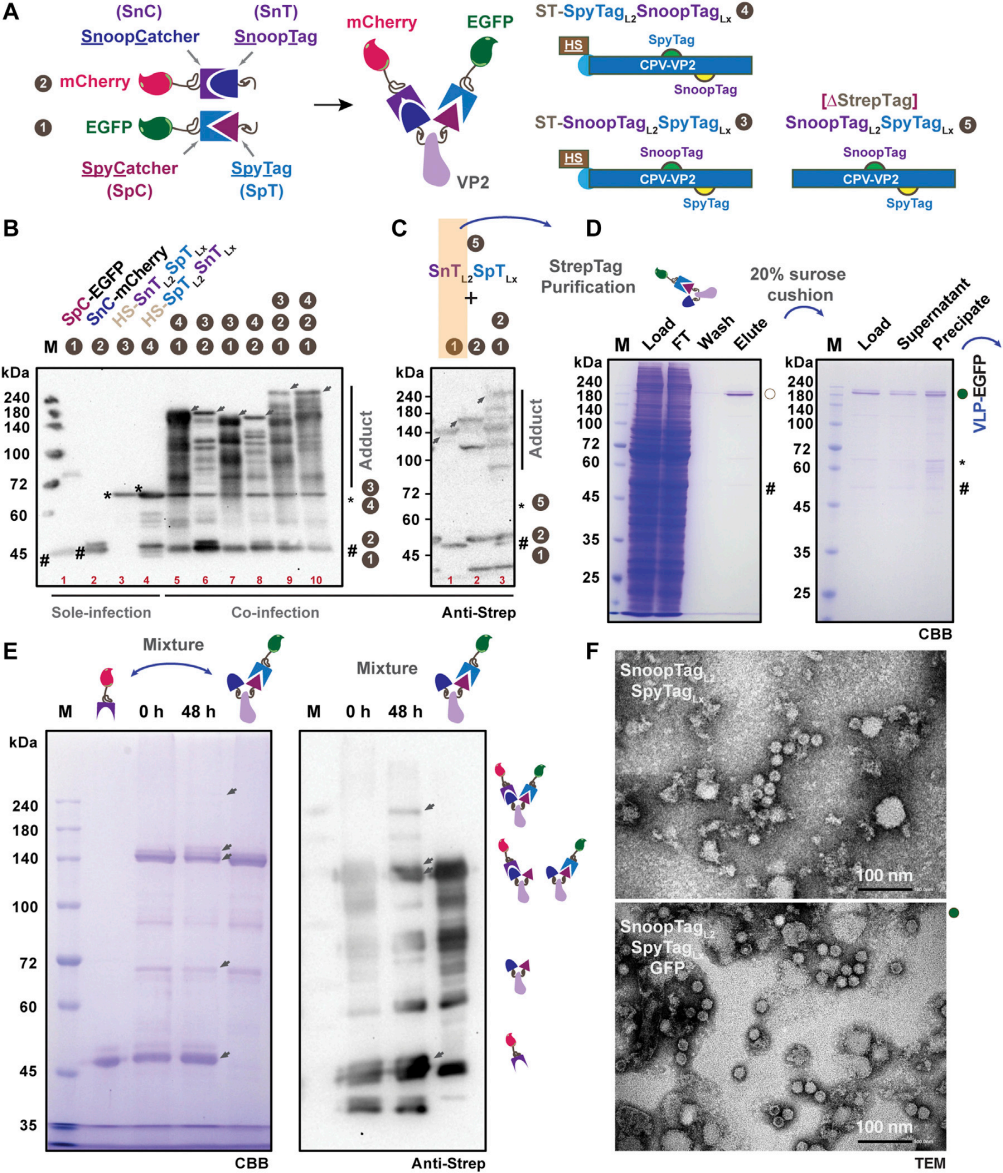
Design and verification of multiple protein display in SpT- and SnT-inserted VP2-derived VLPs. Schematic representation of the specific covalent binding between SpT and SpC, SnT and SnC, respectively. Five constructs, SpC-EGFP 1), SnC-mCherry 2), VP2-SnT_L2_SpT_Lx_ 3), VP2-SpT_L2_SnT_Lx_ 4), VP2-SnT_L2_SpT_Lx_ (∆StrepTag, 5) were designed in this study for multiple protein display **(A)**. Expression verification of those constructs, either in sole, double, or triple infections, was performed in cultured Bm5 cells **(B)**. Upon co-infection, the gradient increased adducts were shown as indicated by arrows. The expression and binding efficiency in Bm5 cells among VP2-SnT_L2_SpT_Lx_ (∆StrepTag, 5), SpC-EGFP (1), and SnC-mCherry were further validated by western blot using the anti-Strep antibody **(C)**. The purification from co-infection of VP2-SnT_L2_SpT_Lx_ (∆StrepTag, 5) and SpC-EGFP (1) in silkworm larvae was conducted, and the elution from *in vivo* binding products was further subjected to ultracentrifugation through a 20% sucrose cushion. The EGFP-displayed VLP (VLP-EGFP) was presented in precipitation **(D)**. *In vitro* binding between purified SnC-mCherry and (SnT_L2_)(SpT_Lx_-SpC-EGFP)] (VLP-EGFP, **(D)** was assayed for 48 h, and the results were demonstrated in SDS-PAGE **(E)**, left panel) and western blot (anti-Strep, **(E)**, right panel). The TEM images for VLP validation are in **(F)** (upper panel: SnT_L2_SpT_Lx_; lower panel: SnT_L2_SpT_Lx_:GFP). Scale bar: 100 nm.

To further realize chemically orthogonal protein display on SpT-VP2-derived VLPs, we introduced SnoopTag-SnoopCatcher (SnT-SnC) chemistry to SpTL2-VP2 as explained in Figure 4A for dual plug-and-display (Brune et al., 2017). Based on this concept, we generated a series of DNA constructs expressing His6-Strep (HS)-tagged VP2 proteins with SnTL2-SpTLx (#3, 72.7 kD), SpTL2-SnTLx (#4, 72.7 kD), HS-tag-free (∆StrepTag) VP2 with ∆StrepTag-STSnTL2-SpTLx (#5, 69.1 kD), as well as SpC-EGFP (#1, 43.8 kD) and SnC-mCherry (#2, 43.5 kD). The expression of each construct, except #5 in cultured Bm5 cells, was verified either in sole infection or co-infection (Figure 4B). The triple coinfections of #1,2,3 (SpC-EGFP, SnC-mCherry, and SnTL2-SpTLx; 160 kD) or #1,2,4 (SpC-EGFP, SnC-mCherry, and SpTL2-SnTLx; 160 kD) resulted in adducts (indicated by arrows) with higher molecular weight than #1,4 (116.5 kD), #2,3 (116.2 kD), #1,3 (116.5 kD), and #2,4 (116.2 kD) double coinfections (Table 2). To facilitate the purification of the antigen-displaying VLP, we further removed fusion tags from SnTL2-SpTLx-VP2, focusing more on the fusion tags from SpC/SnC-tagged antigens. With the HS-tag-free VP2 (#5, 69.1 kD), western blot analysis for samples of co-infection combinations in #1,5 (112.9 kD), #2,5 (112.6 kD), and #1,2,5 (156.4 kD) also showed specific VP2 adducts covalently ligated with either SpC-EGFP (#1), SnC-mCherry (#2), or both (#1 and #2), as indicated by arrows (Figure 4C). These results are similar to the results in Figure 4B. The above results from SpT/SnT double chemistry systems suggest that the ligation among SpT, SpC, SnT, and SnC occurs specifically and orthogonally in silkworm cell-based BEVS.

Aiming at better production of above each protein partner, we then designed to purify SpC-EGFP:SnTL2-SpTLx (#1,5) from the fat body lysates of 10 silkworm larvae. We infected silkworm larvae with a mixture of recombinant baculoviruses at a co-infection rate of about 1:2 (SpC-EGFP:SnTL2-SpTLx). Under this tuned condition, the purification of Strep-tagged SpC-EGFP proteins should ensure a majority of SpC-EGFP:SnTL2-SpTLx-VP2 present in the lysates and the final elution. As expected, the purification results from Figure 4D (left panel) proved that we successfully obtained the ligated adduct of SpC-EGFP:SnTL2-SpTLx-VP2 (VP2-EGFP) with a small fraction of SpC-EGFP presented in the final elution. The VLP assembly for VP2-EGFP-SnTLx (VLP-EGFP with free SnT displayed) was verified in the precipitation after ultracentrifugation through a 20% sucrose cushion (Figure 4D, right panel), which is beyond our expectation since the SpT-L2 inserted chimeric VP2 failed to self-assemble VLPs. To further test the *in vitro* binding capacity of SnT exposed to the VLP surface, we incubated purified VLP-EGFP-SnT and SnC-mCherry proteins for 48 h at 4°C, the results of which are shown in Figure 4E. Specific shifted bands with the highest molecular weight that appeared as confirmed either in CBB staining or western blot are considered as the SnC-mCherry and SpC-EGFP double-displayed VLP products. Moreover, the intact and efficient VLP formation for purified SnTL2-SpTLx-VP2 (bare VLP carrier) and SpC-EGFP:SnTL2-SpTLx-VP2 (SpC-EGFP displaying VLP) were validated by TEM (Figure 4F). Together with the purified VLP-EGFP from 20% sucrose cushion (Figures 4D, F), our study indicates that we successfully developed a VP2-VLP platform for possible multiple protein display based on orthogonal SnT-SnC and SpT-SpC chemistry.

## 4 Discussion

VLP display technology has recently been an attractive approach for antigen/drug delivery against pathogenic infections as vaccines or targeting specific cells *in vivo* as medicines. One application of antigen displaying VLP vaccine is the RTS,S (Mosquirix™) against malaria, which is based on the hepatitis B small surface antigen (HBsAg) particle displaying truncated circumsporozoite protein (CSP) antigen (Rts et al., 2012). Besides the HBsAg-derived VLP carrier, a coat protein CP3 from *Acinetobacter* phage AP205 or other synthetic protein nanocages like i301 have been frequently employed as model VLP carriers for producing antigens displayed VLPs. Most of the terminal positions were used for the genetic fusion of antigens due to their relatively shorter amino acid sequences (Brune et al., 2017; Fuenmayor et al., 2017; Bruun et al., 2018; Qian et al., 2020). Concerning the VP2-derived CPV-LPs or human parvovirus B19, most studies have focused on the fundamental VLP formation and its production using a conventional baculovirus expression system (Brown et al., 1994; Hurtado et al., 1996; Yuan and Parrish, 2001; Sanchez-Rodriguez et al., 2012; Soto-Valerio et al., 2022). Based on the ability of VLP formations from four loop deletion mutations of VP2, Loop 2 (R216-G235, ∆218–233) is considered non-essential compared to the other three loops (Hurtado et al., 1996). The insertion site (S226_G227) from Loop 2 was further proved for its better immunogenicity, possibly because this region locates at the top of the protrusion (Rueda et al., 1999). It has been reported that terminal SpT/SpC-engineered VP2 from human parvovirus B19 can display the desired enzyme and model fluorescent proteins with a limited intensity (Cayetano-Cruz et al., 2018; Soto-Valerio et al., 2022). In this study, we found that N-terminal insertion of SpT does not affect the assemble of VLP (Figure 1F), but the resulting VLPs merely capture free SpC-EGFP for surface display (3.2 ± 1.8/60, ∼5.4%) (Figure 1G; Figure 3G), although a 20% (12/60) display ratio is predicted (Gilbert et al., 2006). We also concluded that VLP formation is maintained with SpT/SnT inserted between S226_G227 from loop 2 (Figure 3H), with which an enhanced VLP displayed was verified (48.2 ± 0.8, ∼80.3%). Since another outer surface residual, T391_G392 (Loop X), has been simulated in an earlier study (Xie and Chapman, 1996), we investigated if it is suitable for SpT/SnT insertion. As shown in Figure 2, VLP formation seems disturbed after SpT was inserted into Loop X. However, it is interesting to observe that the SnTL2-SpTLx double insertion variant formed intact VLPs (Figure 4F). This paradox could be explained partly by the different composition of amino acids of SpT (**VP**T**IVMV**D**A**YKRYK, Hydrophobic amino acids in bold: 50%) and SnT (K**LG**D**I**E**FI**K**V**NK, Hydrophobic: 41.67%), which eventually brings back the correct structural formation of VLP. Further screening of SpT/SnT point mutations, like the one from SpT003 (SpT mutant enhancing reaction rate), could be performed to investigate if VLP formation can be improved in the SpTL2-VP2 variant (Keeble et al., 2019; Vester et al., 2022).

Dual or multiple antigens-displayed VLPs hold advantages over single antigen-bearing VLPs or mixed ones, not only due to the low complexity of dual-displayed VLPs but also when there is a need for synergic protections from multiple antigens. For example, against different stages of a parasite’s life cycle, different strains or serotypes of a parasite, or even different parasites (Brune et al., 2017; Nooraei et al., 2021). It is significant to achieve efficient specific and programable conjugation among purified SpC/SnC-decorated model proteins and SpT/SnT-displaying VLPs, as demonstrated in Figures 3, 4. Similar to AP205-CP3- and HBsAg-derived VLP display systems, the successful development of VLPs displaying double SnTL2-SpTLx superglues allows a synthetic dual play-and-display approach on the VP2-derived VLP platform by directly mixing SpC/SnC-tagged antigens from infectious pathogens like *Plasmodium falciparum* or COVID-19 (Brune et al., 2016; Janitzek et al., 2016; Leneghan et al., 2017; Kim et al., 2019; Marini et al., 2019). Further optimizations could be performed regarding this. 1) *in vitro* binding conditions, including tuning pH and buffer composition since the intensity of double antigen was still not very satisfactory (Fierer et al., 2014); 2) continuous search for an alternative insertion site structurally suitable for display without VLP disruptions (Hurtado et al., 1996; Xie and Chapman, 1996; Li et al., 2014; Biela et al., 2022). Besides, we have to admit that it is also worthwhile to verify the antigen VLP-displaying status using DLS with proper controls and/or immuno-TEM with antigen-specific antibodies as further experiments.

Recently, the BEVS platform has been dramatically improved for both protein expression levels and protein complexes (van Oers et al., 2015; Chambers et al., 2018; Biela et al., 2022), such as GoldenBac (Neuhold et al., 2020), MultiBac (Berger et al., 2004), SpyBEVS (Xu et al., 2019), and Silkworm-BEVS (Kato et al., 2010; Mitsudome et al., 2014). Among the various POIs expressed from BEVS, VLP products have gained much interest since they are usually difficult to be expressed in prokaryotic cells as self-assembled VLPs (Minkner and Park, 2018). To date, there is only limited literature reporting SpC/SpT- or SnC/SnT-tagged proteins produced from cultured insect cells or larvae-based BEVS for protein/antigen conjugations (Xu et al., 2019), Spy-VLP vaccines (Janitzek et al., 2016; Thrane, et al., 2016; Lampinen, et al., 2021; Chevillard et al., 2022), antibody engineering (Akiba et al., 2020), and single-virus tracking (Ke et al., 2018). Notably, those insect cell-derived specific antigen-displaying Spy-VLP vaccines using AP205, norovirus VP1 or adenovirus ADDomer showed satisfactory immunogenic responses in tested animal models targeting malaria (Thrane, et al., 2016), influenza virus (Lampinen, et al., 2021), or SARS-CoV-2 (Chevillard et al., 2022). In the current study, we employed silkworm-SpyBEVS for developing an antigen display VLP platform, where capsid VP2 from non-enveloped CPV was used as a model VLP and further engineered with SpT/SnT. Our study is the first demonstration that the SpT/SnT double engineered VP2 variants enabled an orthogonal conjugation and display of multiply antigens on defined surface regions of VLPs. Further investigations will be focused to verify if a multivalent immunogenic response can be induced *in vivo*. The VP2-derived VLPs were obtained in mg scale per 10 silkworm larvae and self-assembled to an intact structural formation, ensuring convincible VLP display results *in vitro* and *in vivo via* co-infection with the recombinant baculovirus mixture, as demonstrated in Figure 2D; Figures 4B, C. This approach is remarkably efficient for producing protein complexes and timesaving for verifying *in vivo* enzymatic reactions like specific protease (Xu et al., 2022) and the covalent protein conjugation in this study (Xu et al., 2019). It still requires further consideration to apply coinfection or *in vitro* assembly in a case-dependent manner since there is a potential risk for the coupled VP2-antigen protein complex not to be self-assembled into VLP, which is also true for validating the status of each antigen display on VLP surfaces.

Moreover, since the proof-of-concept investigations can be tested in insect cells (e.g., Sf9 or Bm5 cells) at a small-scale level, the silkworm-BEVS can be more cost-effective when applied at an industrial level for scale-up production. Because each silkworm larva can be considered an independent bioreactor, the protein production industrial line could be further integrated with the local silkworm-related agriculture business to reach better manufacturing standards and industry regulations. Automatic silkworm feeding, viral infection, and silkworm dissecting for isolating fat body tissues also need to be established in the future. In addition, it is also worthwhile to perform a comprehensive screening of silkworm larvae to achieve a higher expression level of POI, such as the VP2 protein in this study, for future applications when a large amount of proteins are required (Masuda et al., 2015; Hino et al., 2016). Other VLPs of interest already developed in BEVS could also take advantage of our strategy for plug-and-displayable VLP purposes. It would be exciting for those needing more than one structural protein to assemble VLPs correctly, either non-enveloped or enveloped viruses, such as Rotavirus and Rous sarcoma virus (Minkner and Park, 2018; Kato et al., 2022). As shown in Figure 4, we also successfully purified EGFP-displayed VLPs from fusion tag-free and the SpT/SnT double-tagged VP2 variant. This strategy will allow for application in subsequent immunization in animal experiments directly without additional purification removing fusion tags of antigens.

## Data Availability

The original contributions presented in the study are included in the article/supplementary material, further inquiries can be directed to the corresponding author.
